# Sub-picosecond extraction of hot carriers in black phosphorus

**DOI:** 10.1038/s41467-026-72892-w

**Published:** 2026-05-13

**Authors:** Katsumasa Yoshioka, Taro Wakamura, Takuya Okamoto, Norio Kumada

**Affiliations:** Basic Research Laboratories, NTT, Inc., 3-1 Morinosato-Wakamiya, Atsugi, Japan

**Keywords:** Nanophotonics and plasmonics, Ultrafast photonics, Photonic devices

## Abstract

Harvesting hot carriers before they lose energy to the lattice is a critical route toward surpassing the conventional thermodynamic limit in optical-to-electrical (O-E) conversion. However, photocurrent from such hot carriers has remained challenging to directly detect because they equilibrate on picosecond timescales, outpacing conventional electronic measurement. Here, by employing terahertz electronics with sub-picosecond temporal resolution, we directly monitor ultrafast O-E conversion in black phosphorus (BP). Photoexcitation near the metal contact under zero source-drain bias generates an ultrafast photocurrent with a decay time of ~ 400 fs—orders of magnitude faster than the typical sub-nanosecond energy relaxation in BP—demonstrating a measured 3 dB bandwidth of 260 GHz with an intrinsic limit of ~ 600 GHz. Notably, this photocurrent flows via energetic holes toward the contact electrode, regardless of the equilibrium carrier type. We propose super-diffusive hot-carrier transport as the microscopic origin of the ultrafast photocurrent. Furthermore, we show that the ultrafast hot-carrier contribution can coexist with the much slower cold-carrier contribution based on the photovoltaic effect, demonstrating that hot carriers can be harvested without discarding lower-energy carriers. These findings highlight the potential of sub-picosecond hot-carrier extraction to expand the O-E conversion bandwidth without sacrificing efficiency, bridging fundamental hot-carrier physics with ultrahigh-speed technological applications.

## Abstract (web summary)

Here, the authors observe the excitation of ultrafast photocurrents in black phosphorus flakes with decay times of ~ 400 fs (3-dB bandwidth of 260 GHz), showing the potential of this 2D material for sub-picosecond hot-carrier extraction and high-speed optoelectronic devices.

## Introduction

Hot-carrier extraction is increasingly viewed as a key strategy to circumvent the Shockley-Queisser (SQ) efficiency limit in photovoltaic devices^1–5^ while also enabling ultrafast optical-to-electrical (O-E) conversion for high-speed data communication^6–9^. The underlying challenge lies in the energy loss on a picosecond timescale, which makes hot carriers difficult to capture and utilize in a practical device. To address this, two-dimensional (2D) van der Waals materials have attracted significant attention^5,10–24^ due to their strong quantum confinement and the design flexibility afforded by heterostructuring, both of which can enhance hot-carrier effects. Ultrafast optical spectroscopic studies in these materials have revealed key phenomena, such as efficient carrier multiplication^10,11,23^, tunable decay rates and pathways^13,20,24^, and interlayer hot-carrier generation and transport^12,18,21,22^. However, such optical techniques cannot capture the actual photocurrent, leaving the ultrafast extraction process at metallic electrodes—the critical step for O-E conversion—largely unresolved.

Among 2D semiconductors, black phosphorus (BP) offers a tunable bandgap, anisotropic carrier transport, and high mobility^25,26^ (up to ~10,000 cm^2 ^V^-1^s^-1^). These properties have enabled various photodetector (PD) architectures^27–39^, including broadband^27^, polarization-sensitive^35^, plasmonically enhanced^36,38^, and waveguide-integrated^34,37,38^ designs. In parallel, combining BP with transition metal dichalcogenides (TMDCs) has yielded p-n junction^29,39^ and bulk photovoltaic^30,31^ PDs. Although ultrafast optical measurements show exceptional photocarrier diffusion^40,41^ and multiplication^42^ in BP, the fastest electrically measured 3 dB bandwidth remains limited to ~3 GHz^34^. This indicates that the practical exploitation of hot-carrier effects has been hindered, as the detection of hot-carrier extraction from an electrode requires sub-picosecond electrical readout, which is well beyond the capabilities of conventional electronics.

Here, we integrate BP with an on‑chip, laser‑triggered photoconductive (PC) switch^8,43–48^, enabling direct access to the previously unresolved ultrafast photocurrent extraction step at the electrode. Under zero bias, the photocurrent decays within ~400 fs, implying an intrinsic 3 dB bandwidth of ~600 GHz and highlighting the importance of hot-carrier extraction. In a gate-tunable device architecture, we identify both an ultrafast hot-carrier^49–53^ component that consistently drives energetic holes to the electrode and a slower cold-carrier component whose polarity depends on doping via the photovoltaic (PV) effect. By harvesting both ultrafast hot and slower cold carriers, our strategy may enable femtosecond optoelectronic devices and enhance energy harvesting efficiency.

## Results

### Experimental set-up

**Fig. 1:**
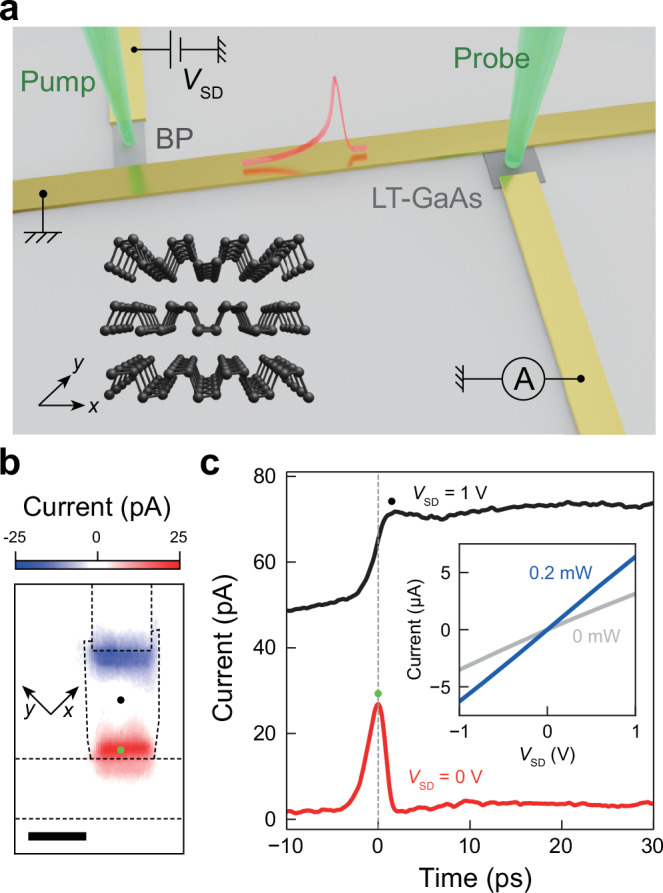
Set-up for ultrafast photocurrent readout. **a** Schematic of the device structure. The black phosphorus (BP) flake is connected to a Goubau line leading to an low-temperature-grown GaAs photoconductive (LT-GaAs PC) switch. A pump-probe method captures the photocurrent waveform in the time domain. The inset shows BP’s crystal structure with *x* (armchair) and *y* (zigzag) axes. **b** Scanning photocurrent map at zero source-drain bias (*V*_SD_ = 0 V), obtained at the peak of transient photocurrent in **c**. Scale bar, 10 µm. The black dashed line outlines the sample structure. (**c**) Temporal profiles of the photocurrent with different pump position and bias conditions. Black and green circles in (**b**) mark the pump spots. The inset shows current-voltage (*I*-*V*) curves with and without the pump beam at the center position (black circle in **b**). BP thickness: 22 nm.

We used three samples with different waveguide and gate structures. The thickness of BP varies between 15 and 25 nm and is indicated in each figure caption. The BP crystal orientation was determined using polarization-resolved Raman spectroscopy (Supplementary Section I) and is noted in the respective figures. We begin with the most straightforward device architecture: a mechanically exfoliated BP flake on a sapphire substrate connected to a low-temperature-grown GaAs (LT-GaAs) PC switch via a 10-µm-wide Goubau-line waveguide (Fig. 1a). A femtosecond laser (280 fs pulse width, 517 nm center wavelength, 16.8 MHz repetition) is split into pump and probe beams: the pump excites the BP, while the probe triggers the PC switch at adjustable delays, enabling photocurrent waveform measurement in the time-domain with sub-picosecond resolution. The pump is tightly focused to perform scanning photocurrent microscopy. All measurements were performed at room temperature.

Figure 1b shows a spatial photocurrent map at zero source-drain bias (*V*_SD_ = 0 V) at zero probe beam delay. Photocurrent appears only near the two metallic contacts with opposite signs, similar to previous reports^30,32,54^. Throughout this work, positive photocurrent denotes flow along the waveguide toward the PC switch. Figure 1c compares time-domain photocurrent signals at different excitation spots and *V*_SD_. As is evident from Fig. 1b, when the center of the flake is excited, no photocurrent is generated at *V*_SD_ = 0. At a finite bias *V*_SD_ = 1 V, a slow photocurrent with no clear decay within 30 ps appears, consistent with the sub-nanosecond energy relaxation reported earlier^40–42,55–58^. The finite offset (~50 pA) before the pump pulse arrives implies incomplete carrier decay between repetitive laser pulses. Increased current flow by photoexcitation confirms a standard PC effect, as shown in the inset’s *I*-*V* data.

In stark contrast, exciting the contact edge (green circle in Fig. 1b) at *V*_SD_ = 0 V generates an ultrafast photocurrent with a full-width at half-maximum (FWHM) of only 2.2 ps, marking the fastest BP PD response to date. Its peak precedes the slower signal (black trace) by ~2 ps, indicating near-instantaneous extraction from the electrode. When applying a *V*_SD_ = ± 1 V, a slow PC response is superimposed on the fast signal (Supplementary Fig. 2 in the Supplementary Information), demonstrating that the two photocurrent responses originate from distinct O-E conversion mechanisms. Observation of this ultrafast response is made possible by our approach, which directly measures the photocurrent extracted from the contact with sub-picosecond time resolution. The remainder of this paper explores the physical origin and intrinsic dynamics of this ultrafast photocurrent.

### Origin of ultrafast zero-bias photocurrent

**Fig. 2:**
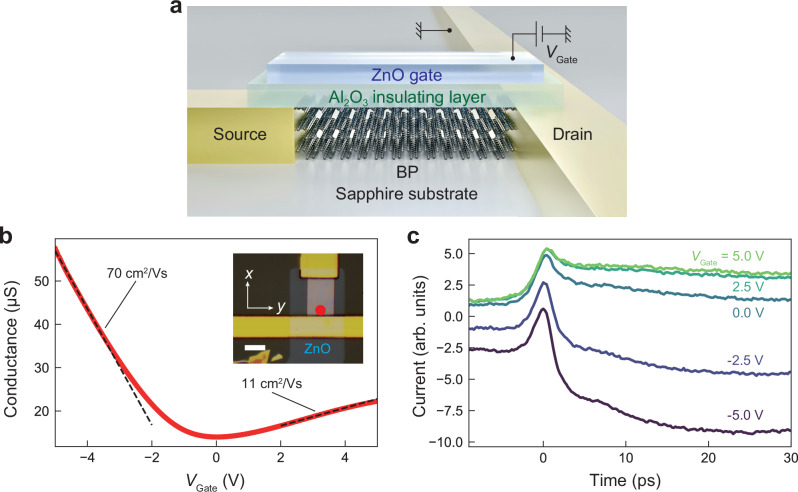
Carrier density dependence of photocurrent. **a** Schematic of a BP flake with a ZnO top gate, minimizing the resistance-capacitance (RC) time constant and permitting gate control of carrier density. **b** BP conductance as a function of gate voltage (*V*_Gate_). Dashed black lines indicate the best linear fits used to determine the field-effect mobility. The inset shows an optical micrograph (scale bar, 10 µm), with the red circle marking the pump spot. **c** Temporal profiles of the zero-bias photocurrent at the contact edge for different *V*_Gate_. BP thickness: 25 nm.

To elucidate the origin of the ultrafast photocurrent, we fabricated a top-gated BP device and examined how carrier density affects the measured signals (Fig. 2a). We used zinc oxide (ZnO) as the top gate electrode to minimize the RC time constant from gate capacitance^8,44,59^. Figure 2b plots BP conductance versus gate voltage *V*_Gate_. The charge neutrality point is located around 0 V, indicating that BP is hole-doped at negative *V*_Gate_ and electron-doped at positive *V*_Gate_. The field-effect mobility in the hole-doped regime (70 cm^2^/Vs) is higher than that in the electron-doped regime (11 cm^2^/Vs), which agrees with previous studies^25,26,60^.

Figure 2c presents the photocurrent at *V*_SD_ = 0 V with the pump beam focused near the contact edge under various *V*_Gate_ values. An ultrafast pulse as in Fig. 1c appears for all the traces, but a slower component decaying over tens of picoseconds also arises, creating an offset before the pump arrives. This slow component becomes more pronounced at larger doping (*V*_Gate_ = ±5 V), which explains the worse visibility in the ungated device. Because these fast and slow signals respond differently to doping, they likely arise from distinct photocurrent generation mechanisms. In BP, multiple decay timescales have been observed by ultrafast optical spectroscopies, spanning tens of femtoseconds^55,61^, sub- to a few picoseconds^42,55–58,62^, and extending up to several hundred picoseconds^40–42,55–58^. The fastest response is associated with the thermalization time, reflecting how rapidly a Fermi-Dirac distribution forms through carrier-carrier scattering, whereas the slowest response corresponds to the recombination of cold carriers that exist at the band edge. An intermediate picosecond response is attributed to hot-carrier cooling, where an elevated carrier temperature decreases via carrier-phonon scattering. In our measurements, the observed fast signals fall within the hot-carrier regime, whereas the slow signals correspond to the cold-carrier regime. We note that the thermalization time, which mainly determines the initial rise of the photocurrent, is not resolved within our experimental bandwidth (see Supplementary Section IV for details).

**Fig. 3:**
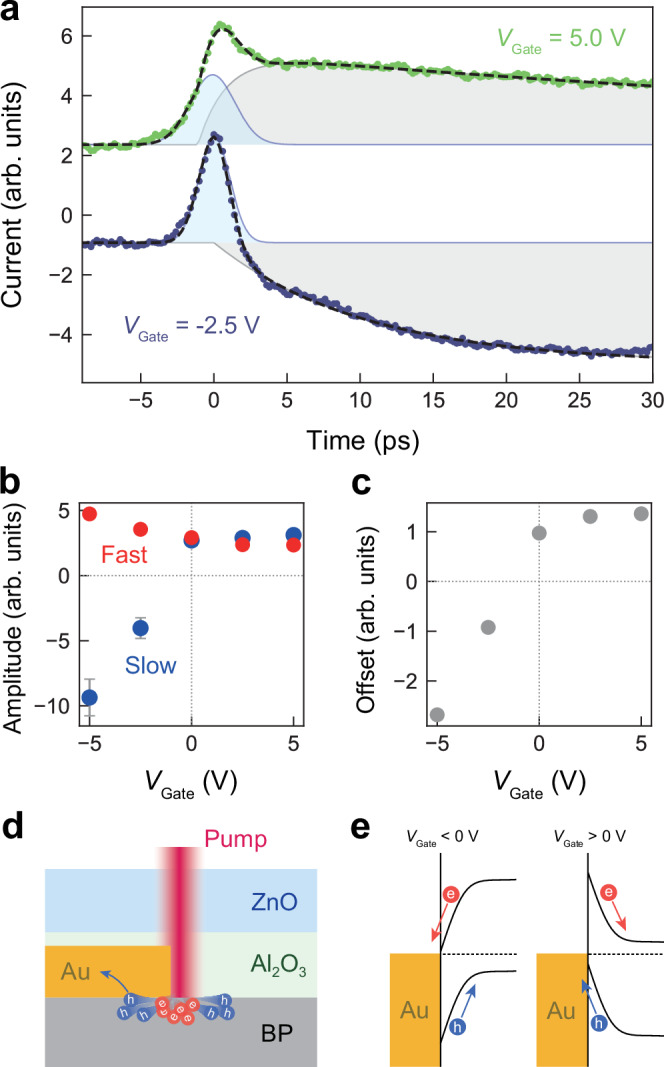
Photocurrent generation mechanisms. **a** Experimental (colored) and fitted (dashed black) photocurrent waveforms at *V*_Gate_ = −2.5 V and +5.0 V, with Gaussian (fast, light blue) and exponential (slow, gray) components. The trace for *V*_Gate_ = +5.0 V is offset by 1.0 for clarity. **b** Fitted amplitudes of the fast and slow components as a function of *V*_Gate_. Error bars represent the standard errors of the fitted parameters. **c** Fitted offset as a function of *V*_Gate_. (**d**) Schematic of the proposed fast photocurrent origin (super-diffusion effect). **e** Schematic of the slow photocurrent origin (photovoltaic (PV) effect under built-in electric field), shown using a band diagram of the Schottky junction. Red and blue circles represent holes and electrons, respectively, and arrows indicate their direction of motion.

To separate these components, we fitted the experimental traces with a sum of a Gaussian (fast) and an exponential rise-decay (slow) function (Supplementary Section III), as illustrated in Fig. 3a. The data are well replicated by this two-term model. Figure 3b shows the fitted amplitudes of each component as a function of *V*_Gate_. The fast component remains positive for all *V*_Gate_, indicating a consistent flow of photoexcited holes to the contact regardless of whether BP is hole- or electron-doped. In contrast, the slow response changes sign with *V*_Gate_: electrons flow to the contact at negative *V*_Gate_, while holes flow to the contact at positive *V*_Gate_.

These observations suggest that the consistent ultrafast hole flow arises from super-diffusion^49–53^ of hot carriers. Immediately after photoexcitation, carriers expand rapidly with a diffusion constant up to ~1000 times higher^49^ than that of cold carriers, owing to their elevated effective temperature and kinetic energy. This super-diffusion ends once carriers cool^49–53^, matching the picosecond dynamics observed near the contact. Two independent considerations help explain why holes dominate the extracted current. First, BP exhibits higher hole mobility than electron mobility^25,26,60^, consistent with our gate-dependent transport data in Fig. 2b. Second, ultrafast electron microscopy^41^ shows that photoexcited holes diffuse in-plane, whereas electrons diffuse out of plane under the influence of the BP’s surface potential. Together, these factors are expected to favor hole collection regardless of the equilibrium doping type and distinguish the response from conventional PV or photothermoelectric (PTE) effects. Consequently, energetic holes are consistently extracted at the contact, consistent with super-diffusive transport (Fig. 3d). Additional analyses excluding PV, PTE, and displacement-current^63^ contributions to the fast component are provided in Supplementary Section V.

On the other hand, the change in the sign of the slow response is consistent with a PV mechanism at the Schottky junction formed at the metal-semiconductor interface^32,54^ (Fig. 3e). After the super-diffusion process, the remaining carriers cool to the band edge and are then driven to the electrode by the built-in field, with a possible additional contribution from barrier-height modulation^64^, both of which depend on *V*_Gate_. The offset before the pump laser pulse (Fig. 3c) follows the same trend as the slow-component amplitude, indicating a long carrier lifetime (>59.5 ns) that rules out a PTE origin for this slow signal.

In addition, we investigated the pump-wavelength dependence using 517 and 1035 nm excitation (see Supplementary Section VI for details). The qualitative features of both the fast and slow responses remain unchanged across wavelengths, indicating that they originate from intrinsic transport processes of photoexcited carriers in BP and are not specific to 517 nm excitation.

Both fast and slow photocurrent components in a gate-tunable device need to be controlled to understand photocurrent generation mechanisms in BP. For practical applications, the optimal gate bias can be selected on the basis of the desired outcome: strong doping for maximum energy harvesting, or minimal doping (near the flat band at the Schottky junction) for ultrafast O-E conversion without significant slow response.

### Intrinsic dynamics of hot-carrier extraction

**Fig. 4:**
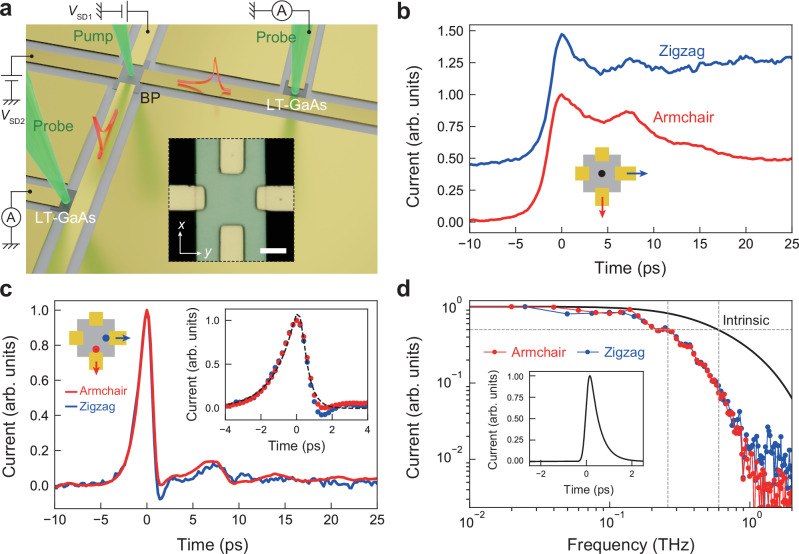
Intrinsic dynamics and crystal orientation dependence of photocurrent. **a** Schematic of the device structure. A BP flake is connected orthogonally to two coplanar waveguides (CPWs) for measuring armchair (*x*) and zigzag (*y*) directions. The inset shows an optical micrograph (scale bar, 10 µm). **b** Temporal profiles of the biased photocurrent for zigzag (*V*_SD2_ = 1 V) and armchair (*V*_SD1_ = 1 V) directions, normalized to the armchair peak. The inset schematically illustrates the corresponding photocurrent extraction directions with arrows. **c** Temporal profiles of the zero-bias photocurrent at the contact edge for each orientation, normalized to the peak after subtracting a minor offset. The inset shows a magnified view near the peak position. The dashed curve shows the best fit to the armchair trace obtained using the analytical convolution model. **d** Fourier-transformed spectra obtained from the waveforms in (**c**) (colored traces) and estimated intrinsic response (black curve). Vertical dashed lines indicate 3 dB bandwidth of 260 and 600 GHz. The inset shows estimated intrinsic photocurrent waveform. BP thickness: 15 nm.

Next, we investigate intrinsic dynamics of hot-carrier extraction and the effect of crystal anisotropy of BP on it. To this end, we fabricated a device with orthogonal coplanar waveguides (CPWs) aligned to the armchair (*x*) and zigzag (*y*) directions of the BP crystal (Fig. 4a). Compared to the Goubau line, CPWs reduce waveguide dispersion, enabling intrinsic photocurrent dynamics to be more accurately measured. The CPW comprises a 10-μm-wide center conductor flanked by two ground planes, each separated from the center conductor by a 7-μm gap. By selecting which PC switch receives the probe beam, we can capture the photocurrent along either the armchair or zigzag directions.

Figure 4b displays photocurrent waveforms under a 1 V bias applied to the electrode opposite to the readout electrode with the center spot excitation, driving a PC response. Along the zigzag direction, the response remains slow with no clear decay within the measurement window, similar to Fig. 1c (black trace). A small peak at ~7 ps arises from multiple reflections between the BP contact and the PC switch, which appears at every measurement configuration in this CPW device. In contrast, the armchair orientation exhibits a faster decay (~30 ps) with negligible offset. This difference aligns with the PC effect picture, where carriers transit more rapidly along the higher-mobility armchair direction^25,40,41^, shortening the observed decay.

Figure 4c highlights zero-bias photocurrent at the contact edge for both orientations. Unlike the biased case, the ultrafast waveforms appear nearly identical in armchair and zigzag directions, indicating that mobility has little effect on the photocurrent decay. This insensitivity aligns with the super-diffusion picture, in which decay dynamics are governed primarily by energy relaxation of hot carriers. Mobility is determined by momentum relaxation, whereas hot-carrier cooling is governed by energy relaxation via optical-phonon emission. As a result, the cooling time is not expected to directly reflect the transport anisotropy encoded in the mobility. Consistent with this picture, previous ultrafast optical studies on BP have reported nearly identical sub-picosecond hot-carrier decay dynamics along the armchair and zigzag directions^56,62^, despite pronounced anisotropy in mobility. In contrast to the nearly identical decay dynamics, the photocurrent amplitude along the armchair direction is larger than that along the zigzag direction (Supplementary Fig. 7a in the Supplementary Information). We find that this combination of direction-independent decay dynamics and anisotropic amplitude can be quantitatively reproduced by a super-diffusion model^49^ using known material parameters and a hot-carrier enhancement factor for the diffusion constant (see Supplementary Section VII for details).

The FWHM of the ultrafast response is ~1.7 ps, shorter than the ~2.2 ps from the Goubau-line device (Fig. 1c, red trace) due to reduced dispersion. It nevertheless remains longer than the CPW’s shortest feasible pulse (~1.2 ps^44^), suggesting that residual dispersion in the CPW does not dominate the measured response. Consequently, the measured photocurrent *j*_meas_(*t*) is the convolution of the photocurrent at the BP (*j*_gen_(*t*)) and the response of the detection PC switch (*j*_det_(-*t*)), expressed as *j*_meas_(*t*) ∝ *j*_gen_(*t*) ∗ *j*_det_(-*t*), where *t* denotes the pump-probe delay^45^. By fitting an analytical model that also accounts for the femtosecond laser pulse duration^45^ (Supplementary Section IV), we extract an intrinsic photocurrent decay constant of 410 ± 30 fs, which is in good agreement with our simulated super-diffusive hot-carrier extraction (Supplementary Fig. 7c in the Supplementary Information).

The 3 dB bandwidth of our BP PD estimated by Fourier-transforming the measured time-domain waveform is approximately 260 GHz (Fig. 4d). Furthermore, by removing the bandwidth limit imposed by the detection PC switch, we infer an intrinsic 3 dB bandwidth near 600 GHz. This bandwidth is derived by Fourier-transforming the waveform (inset of Fig. 4d) after removing the convolution effect of *j*_det_(-*t*) (Supplementary Section IV). These values are nearly two orders of magnitude higher than the previously reported maximum of ~3 GHz achieved by the PV effect^34^, and the inferred intrinsic value reaches the range of the fastest graphene PDs reported to date (~500 GHz^9^). Our O-E conversion response is also substantially faster than the 68–488 ps intrinsic photocurrent response times derived under identical excitation conditions (contact-edge excitation, zero bias) using a photocurrent autocorrelation technique^31^, which utilizes photocurrent saturation effects between two consecutive femtosecond laser pulses. In that approach, the decay time reflects the overall energy relaxation of photoexcited carriers, including recombining cold carriers as well as thermalizing and cooling hot carriers. In contrast, our time-resolved measurements directly isolate the ultrafast photocurrent driven by hot carriers, which precedes and is distinct from any slower cold-carrier effects. This distinction is consistent with a picture in which hot carriers are extracted through super-diffusive transport, whereas cold carriers are collected through built-in electric fields. Without direct sub-picosecond readout at the contact, this hot-carrier contribution would remain unresolved.

## Discussion

In this study, we identified an ultrafast photocurrent in BP with a decay time of approximately 400 fs, a measured 3 dB bandwidth of 260 GHz, and an estimated intrinsic bandwidth of 600 GHz. The remarkable features of this response—including its sub-picosecond lifetime, direction-independent decay dynamics, anisotropic amplitude, and consistent flow of photoexcited holes irrespective of doping type—are most convincingly explained by a super-diffusion mechanism^49–53^. Nevertheless, further experimental and theoretical works will be required to establish the microscopic origin of the observed photocurrent more conclusively and to exclude other possible mechanisms. Our direct readout of ultrafast photocurrent, which captures non-local hot-carrier transport at the semiconductor-metal interface, proved essential for revealing this process. While conventional optical pump-probe techniques provide valuable insights into energy relaxation near the excitation spot, they do not directly access the spatial transport and extraction of hot carriers at contact interfaces, which is an essential aspect of ultrafast O-E conversion.

Super-diffusion of photoexcited carriers has been observed in a wide range of materials (including semiconductor^49^, metal^50^, perovskite^51^, polymer^65^, and TMDCs^52,53^) by ultrafast microscopy methods. While a transient dipole formed by asymmetric carrier distribution has been used to emit broadband THz radiation via the photo-Dember effect^47,66,67^, the microscopic origin and device implications of super-diffusion are distinct and have not yet been exploited for ultrafast hot-carrier extraction in optoelectronic applications.

Notably, we also observed the coexistence of an ultrafast component consistent with super-diffusive transport and a slower component due to a conventional PV effect, indicating that extracting hot carriers does not inherently reduce overall O-E conversion efficiency by discarding cold carriers. Instead, both energetic and relaxed carriers can be harvested, effectively enhancing the O-E conversion bandwidth and efficiency. From a PD design perspective, super-diffusion can in principle be harnessed by placing the metallic contact close to the photoexcitation region, enabling carriers to be collected before they cool. While we employed a basic source-drain channel layout to elucidate fundamental aspects of hot-carrier extraction, device structures incorporating waveguide integration or metamaterial engineering could amplify this effect by maximizing the fraction of carriers reaching the contact in their hot state.

We believe that these findings introduce a robust strategy for enhancing PD performance and O-E conversion across various material platforms. By merging super-diffusion with existing device principles, the ultrafast extraction of hot carriers can offer a distinct performance advantage, which will be highly beneficial for developing ultrafast optoelectronics.

## Methods

### Device fabrication

PC switches were prepared using a LT-GaAs wafer supplied by BATOP, GmbH. The wafer consisted of a 2.6-μm-thick LT-GaAs surface layer (grown at 300 °C) and a 500-nm-thick Al_0.9_Ga_0.1_As sacrificial layer on a semi-insulating GaAs substrate. After patterning the LT-GaAs layer into 100 × 100 µm squares with a citric acid solution, the sacrificial layer was dissolved in hydrochloric acid. The resulting LT-GaAs chips were then transferred onto a sapphire substrate using a thermoplastic methacrylate copolymer (Elvacite 2552 C, Lucite International) as an adhesive^68^. Residual Elvacite on the sapphire surface was removed with citric acid.

BP was obtained by mechanically exfoliating bulk crystal onto a silica (285 nm)/doped silicon substrates. BP flakes with thicknesses of ~15–25 nm were identified using atomic force microscopy. By using Elvacite, the selected BP flake was picked up and transferred onto the sapphire substrate. Next, Ti/Au waveguides and contacts to the BP were deposited by vacuum evaporation. The entire surface was covered with a 30-nm-thick alumina (Al_2_O_3_) insulating layer grown by atomic layer deposition (ALD). For the ZnO gate device, a 20-nm-thick ZnO top gate (ALD at 140 °C) was patterned on the Al_2_O_3_ layer using photolithography and liftoff. An additional Al_2_O_3_ layer was then deposited to protect the ZnO gate. Finally, to provide electrical access to the waveguide, Al_2_O_3_ on the bonding pads was selectively removed using Miroposit 351 developer. BP conductance (Fig. 2b) was characterized using a standard lock-in based DC conductance measurement.

### On-chip terahertz spectroscopy measurements

A femtosecond laser (Monaco, Coherent, Ltd) was used as the light source, and the second-harmonic wavelength of 517 nm was generated using a beta barium borate crystal. Two orthogonally polarized pump and probe beams were combined by a polarization beam splitter and aligned with a slight displacement to focus them onto the BP and the LT-GaAs PC switch, respectively, through an objective lens^69^. The pump power was 0.2 mW (horizontal polarization), with a spot size of ~1.8 µm (FWHM). The pump-power dependence is discussed in Supplementary Section VI. The position of the pump beam was controlled using a motorized mirror, while the probe beam’s position was kept constant throughout the experiments. The zero-time delay was defined as the peak of the fast super-diffusive photocurrent component. An optical chopper modulated the pump beam at a few hundred hertz for lock-in detection of the terahertz current. All measurements were performed under vacuum to minimize sample degradation and environmental effects.

## Supplementary information

Supplementary Information

Transparent Peer Review file

## Data Availability

Relevant data supporting the key findings of this study are available within the article and the Supplementary Information file. All raw data generated during the current study are available from the corresponding authors upon request.
