# Supplementary material for: Sub-picosecond extraction of hot carriers in black phosphorus
Source: Nat Commun. 2026 May 13;17:6193. doi: 10.1038/s41467-026-72892-w (PMC13369905; doi:10.1038/s41467-026-72892-w)
Supplement: Supplementary file 1 — Supplementary Information [file 41467_2026_72892_MOESM1_ESM.pdf]

Supplementary Information for

Sub-picosecond extraction of hot carriers in black phosphorus

Katsumasa Yoshioka[1,*], Taro Wakamura[1], Takuya Okamoto[1], and Norio Kumada[1]

[1]Basic Research Laboratories, NTT, Inc., 3-1 Morinosato-Wakamiya, Atsugi, 243-0198, Japan

*e-mail: katsumasa.yoshioka@ntt.com

I. Polarization-resolved Raman spectroscopy

To determine the crystal orientation of black phosphorus (BP) in the fabricated devices, we performed polarization-resolved Raman spectroscopy (Supplementary Figure 1). Supplementary Figures 1a and 1d correspond to the ungated Goubau-line device (Fig. 1 in the main text), Supplementary Figures 1b and 1e to the ZnO-gated Goubau-line device

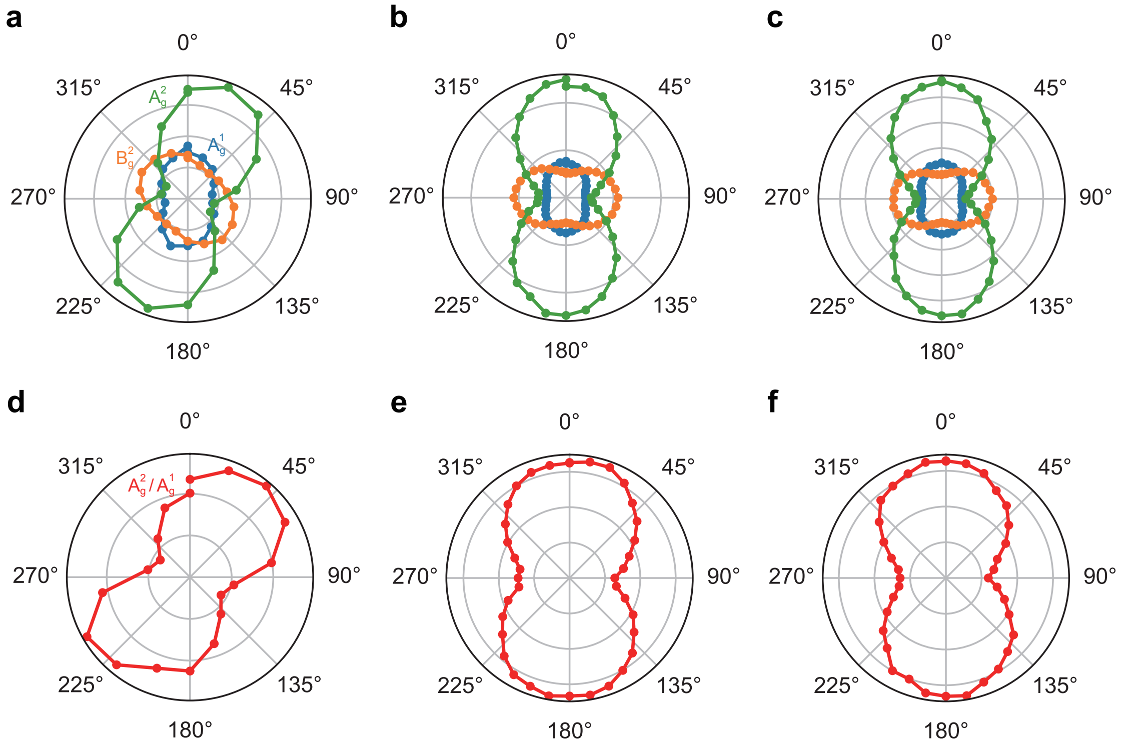

Supplementary Figure 1 | Polarization-resolved Raman spectroscopy. a-c, Intensity of the A_g^1, A_g^2, and B_g^2 Raman modes plotted against polarization angle for the three devices. **d-f**, Raman intensity ratio A_g^2/ A_g^1, used to identify the armchair (x) direction in each device.

(Fig. 2), and Supplementary Figures 1c and 1f to the CPW device (Fig. 4). A linearly polarized 532 nm laser was focused onto the BP through a 100× objective lens, with polarization controlled by a half-wave plate. The intensities of the A_g^1, A_g^2, and B_g^2 Raman modes were extracted by fitting each spectrum to a Lorentzian function.

We define the armchair direction (x) as the angle at which the ratio of the A_g^2 to A_g^1 Raman intensities (A_g^2/A_g^1) is maximized[1,2]. This angle is ~45° for the device in Fig. 1 of the main text and ~0° for the devices in Figs. 2 and 4.

II. Source–drain bias dependence of the contact-edge photocurrent

Supplementary Figure 2 shows the effect of the source–drain bias on the contact-edge photocurrent. Applying V_{SD} = ±1 V to the device shown in Fig. 1 induces a clear slow component driven by the photoconductive effect. This behavior is consistent with the slow photovoltaic response observed under a finite built-in electric field in the absence of a source–drain bias (Fig. 3a,b). These results indicate that the relative contributions of ultrafast and slow photocurrent channels in BP can be tuned by the local band profile, which is controlled by V_{Gate} and V_{SD}, providing useful flexibility for device optimization.

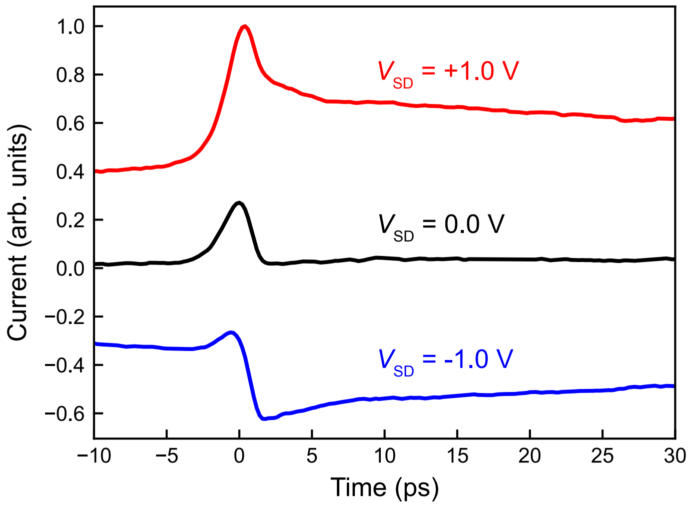

Supplementary Figure 2 | Effect of source–drain bias on slow component. Transient photocurrent profiles measured at the bottom contact edge of the Goubau-line waveguide device (Fig. 1) under different V_{SD} values. The excitation spot is indicated by the green circle in Fig. 1b.

III. Disentangling fast and slow responses

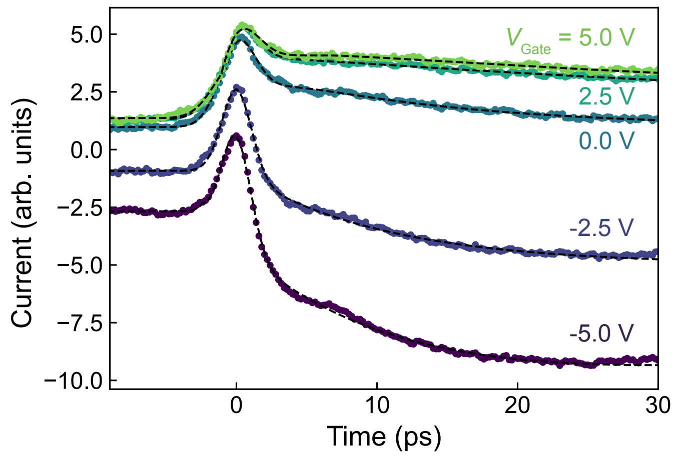

Supplementary Figure 3 | Gate-dependent fast and slow photocurrent. Dashed curves indicate the best fits obtained using the combined Gaussian (fast) and exponential rise–decay (slow) model.

To separate the fast and slow components of the gate-tunable photocurrent (Fig. 2c in the main text), we fit the experimental data using a combined Gaussian (fast) and exponential rise–decay (slow) model:

$$A\exp\left(-\frac{t^2}{2\sigma^2}\right) + B\left(\exp\left[-\frac{t}{\tau_{\mathrm{decay}}}\right] - \exp\left[-\frac{t}{\tau_{\mathrm{rise}}}\right]\right) + C,$$

where A and B are the amplitudes of the fast and slow responses, C is the offset, σ^2 is the variance, and τ_{rise} and τ_{decay} are the rise and decay time constants, respectively. The resulting fits appear in Supplementary Figure 3, while the corresponding fitted values of A, B, and C are shown in Figs. 3b and 3c of the main text.

IV. Analysis of photocurrent decay time

The photocurrent waveform measured in an on-chip geometry can be described as the convolution of generation and detection processes, as developed by P. Zimmermann *et al.*[3] Specifically,

$$j_{\mathrm{meas}}(t) \propto j_{\mathrm{gen}}(t) * j_{\mathrm{det}}(-t),$$

where t denotes the pump-probe delay. In addition, $j_{\mathrm{meas}}(t)$ itself results from convolving the pump-laser excitation $P_{\mathrm{pump}}(t)$ with the carrier density $n_{\mathrm{gen}}(t)$ in the generation switch (BP):

$$j_{\mathrm{gen}}(t) \propto P_{\mathrm{pump}}(t) * n_{\mathrm{gen}}(t).$$

Here, $P_{\mathrm{pump}}(t)$ is modeled by a Gaussian function as

$$P_{\text{pump}}(t) \propto \exp\left(-\frac{t^2}{2\,\sigma_1^2}\right),$$

with its standard deviation σ_1 related to the laser pulse full-width at half-maximum (t_{p1}) by $t_{\text{p1}} = 2\sqrt{2\,\ln(2)}\,\sigma_1$. The function $n_{\text{gen}}(t)$ is taken as an exponential decay with carrier lifetime in BP τ_1 multiplied by a unit step function that is zero before the pump arrives:

$$n_{\text{gen}}(t) \propto \exp\left(-\tfrac{t}{\tau_1}\right)\,\text{UnitStep}(t)\,.$$

Similarly, the carrier density $n_{\text{det}}(t)$ in the detection switch (GaAs) is modeled as

$$n_{\text{det}}(t) \propto \exp\left(-\tfrac{t}{\tau_2}\right)\,\text{UnitStep}(t),$$

where τ_2 is the carrier lifetime in GaAs.

The model assumes a step-like carrier response following excitation, which is reasonable given the short carrier scattering times (tens of femtoseconds) reported in both BP[4,5] and GaAs[6].

Within this framework, the measured photocurrent can be written in analytic form as

$$j_{\text{meas}}(t) = A\left[\exp\left(\frac{\sigma_1^2+\sigma_2^2+2\,t\,\tau_1}{2\,\tau_1^2}\right)\text{erfc}\left(\frac{\sigma_1^2+\sigma_2^2+t\,\tau_1}{\sqrt{2}\,\tau_1\sqrt{\sigma_1^2+\sigma_2^2}}\right) + \exp\left(\frac{\sigma_1^2+\sigma_2^2-2\,t\,\tau_2}{2\,\tau_2^2}\right)\text{erfc}\left(\frac{\sigma_1^2+\sigma_2^2-t\,\tau_2}{\sqrt{2}\,\tau_2\sqrt{\sigma_1^2+\sigma_2^2}}\right)\right],$$

where A is the amplitude, and the index 1 (2) is the generation (detection) switch. To

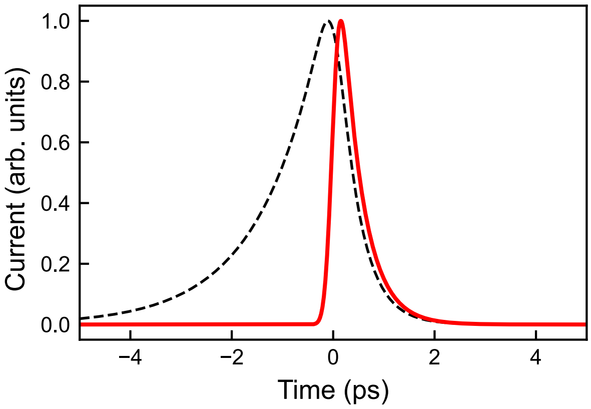

Supplementary Figure 4 | Photocurrent waveforms. The dashed curve shows the best fit to the measured photocurrent using $t_{\text{p1}} = 280$ fs, $\tau_1 = 410$ fs, $t_{\text{p2}} = 280$ fs, and $\tau_2 = 1210$ fs shown in inset of Fig. 4c. The red curve represents the estimated intrinsic photocurrent dynamics obtained by setting $t_{\text{p1}} = 280$ fs, $\tau_1 = 410 \pm 30$ fs, $t_{\text{p2}} = 1$ fs, and $\tau_2 = 1$ fs.

determine the BP photocurrent decay time, we fit the experimental data (inset of Fig. 4c of the main text) by setting $t_{p1} = t_{p2} = 280$ fs, which is measured via an autocorrelation technique. From this fit, we extract a BP carrier lifetime $\tau_1 = 410 \pm 30$ fs and a detection PC switch lifetime $\tau_2 = 1210$ fs $\pm$ 40 fs.

To estimate the intrinsic photocurrent dynamics at the BP-metal interface (inset of Fig. 4d) by removing the bandwidth limit imposed by the detection PC switch, we set $t_{p1} = 280$ fs, $\tau_1 = 410 \pm 30$ fs, $t_{p2} = 1$ fs, and $\tau_2 = 1$ fs. The fitted result and the estimated photocurrent waveform are shown in Supplementary Figure 4.

V. Exclusion of alternative mechanisms for the fast photocurrent component

In this section, we present additional analyses that allow us to unambiguously attribute the fast photocurrent component to super-diffusive in-plane transport of hot holes.

Photothermoelectric (PTE) effect

We evaluated the PTE contribution using the Seebeck coefficient (S_{BP}) extracted from the gate-dependent conductance of BP (Fig. 2b), calculated via the Mott formula[7] (Supplementary Figure 5a). The PTE current based on the calculated Seebeck coefficient $I_{PTE} = S_{BP}(T_{el} - T_0)/R$ (Supplementary Figure 5b) exhibits a polarity reversal between electron- and hole-doped regimes and characteristic peaks at $V_{Gate} \approx \pm 2$ V, consistent with known PTE-driven photodetectors[8,9]. As shown in Supplementary Figure 5c, the experimentally observed fast component never reverses polarity and instead increases monotonically with hole doping, which is entirely inconsistent with PTE, whereas the slow component follows the expected PTE/PV-like trend.

Schottky-junction photovoltaic (PV) / built-in-field effects

At a metal-BP contact, the direction of the built-in electric field reverses depending on whether BP is electron-doped or hole-doped, and its magnitude increases with increasing carrier density. Consequently, the photocurrent induced by the built-in electric field is expected to reverse with the gate polarity and to increase as the system moves away from the charge neutrality point[10]. This behavior matches our slow component but is incompatible with the fast component (Supplementary Figure 5c). Moreover, even the fastest reported PV response in waveguide-integrated BP photodetectors remains limited

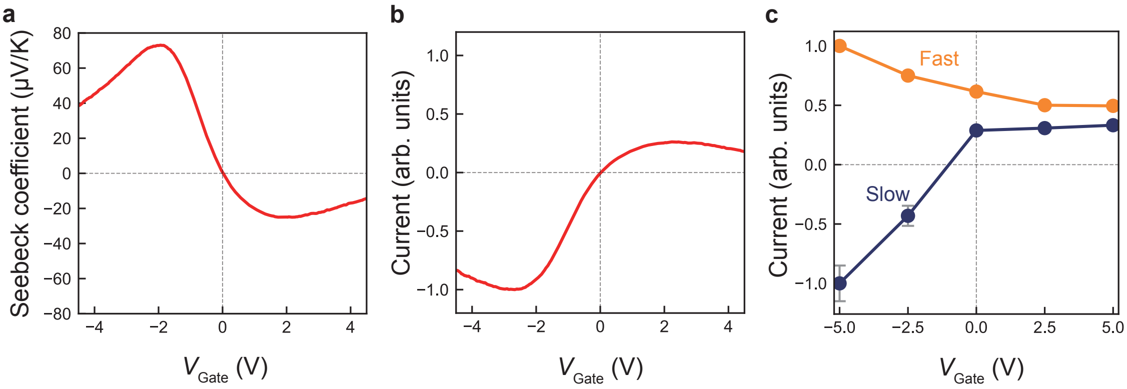

Supplementary Figure 5 | Contribution of photothermoelectric effect. **a**, Calculated Seebeck coefficient using the Mott formula. **b**, Estimated PTE current amplitude derived from the calculated Seebeck coefficient. **c**, Experimentally measured photocurrent amplitudes of the fast and slow components as a function of gate voltage. The amplitudes in (**b**) and (**c**) are normalized for direct comparison. Positive charge flow into the contact is defined as a positive photocurrent.

to the sub-ns regime[11], far slower than the ~2.2 ps transient observed here.

<u>Displacement current</u>

A displacement current $I_{disp} = \varepsilon_0 \varepsilon \partial E/\partial t$[12] could, in principle, contribute at picosecond timescales. However, such a current is determined by the transient change of the built-in electric field and therefore should exhibit the same gate-dependent polarity reversals as PV, entirely inconsistent with the experimentally observed fast component. Thus, the fast component cannot originate from Schottky-related mechanisms.

After excluding PTE and PV contributions based on gate-polarity, functional form, and intrinsic timescale, the mechanism most consistent with all observations is hot-hole extraction enabled by in-plane super-diffusive transport: it naturally accounts for the sub-picosecond lifetime and the consistent flow of photoexcited holes irrespective of doping type. The opposite polarity of the fast component at the two contacts observed in Fig. 1b can also be explained by super-diffusive transport. Since the super-diffusive propagation length (< 1 μm) is much shorter than the channel length (~18 μm), only the electrode closest to the excitation spot contributes to the fast signal, producing a positive photocurrent at one contact and a negative photocurrent at the other.

We note that the suppression of PTE under zero source–drain bias and the dominance of slow PV response in inhomogeneously doped BP have been demonstrated in dual-gated architectures[13], consistent with our findings. The ZnO top-gate structure with on-chip readout in our device enables sub-picosecond electrical detection, allowing the super-diffusive component to be resolved for the first time.

VI. Power and wavelength dependence

We performed additional measurements of the pump-power dependence using both 517 nm and 1035 nm excitation. All measurements were carried out on the same ZnO-gated device shown in Fig. 2 and Fig. 3, which exhibits both fast and slow response channels.

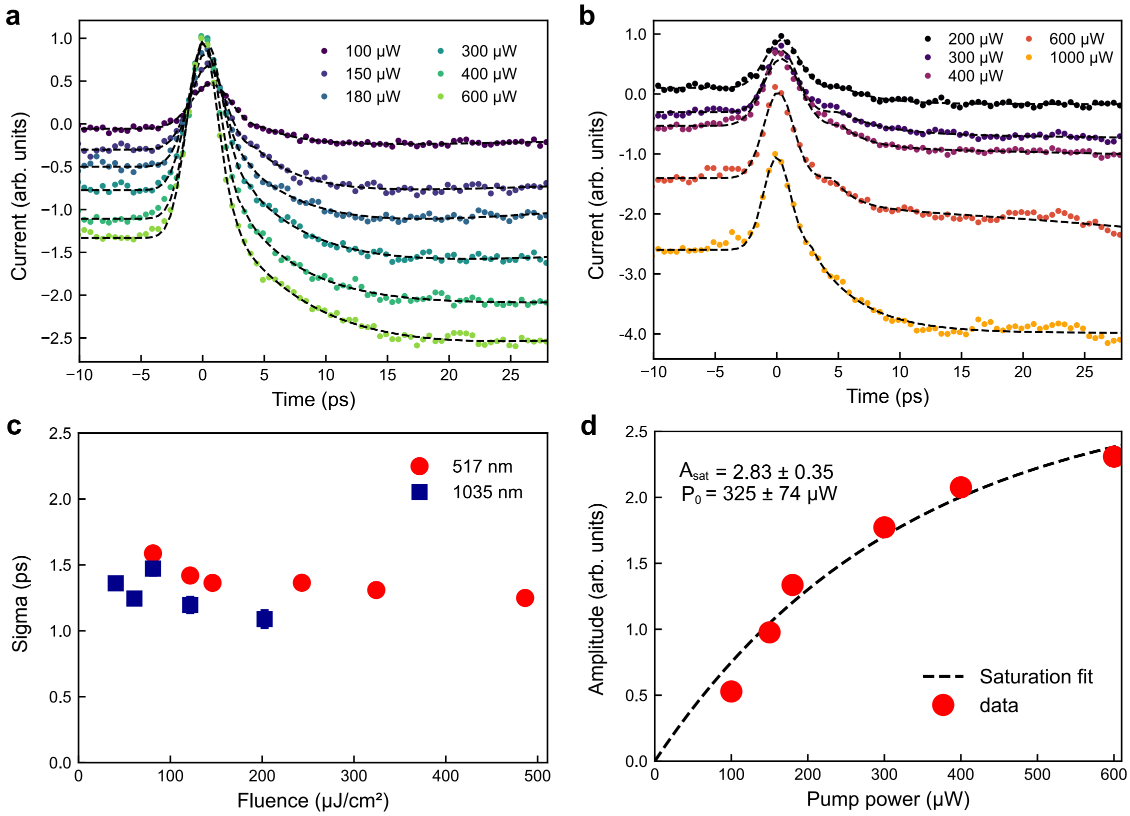

Supplementary Figure 6 | Power and wavelength dependence. a,**b**, Photocurrent transients measured under 517 nm (**a**) and 1035 nm (**b**) excitation with different pump power. Black dashed curves show the best fit obtained using the combined Gaussian (fast) and exponential rise–decay (slow) model. **c**, Extracted Gaussian width σ of the fast component for both wavelengths. **d**, Amplitude of the fast component as a function of pump power (517 nm excitation). Black dashed curve shows the best fit to $A_{\mathrm{sat}}(1 - \exp^{-P/P_0})$.

As shown in Supplementary Figure 6a–b, the photocurrent transients at the two wavelengths exhibit the same qualitative two-component structure, a picosecond Gaussian-like peak followed by a much slower exponential tail, indicating that the coexistence of fast and slow channels is intrinsic to BP and not specific to a particular excitation energy. Notably, the fast component is more prominent for 517 nm excitation. This is consistent with the super-diffusion picture, where higher excess carrier energy leads to stronger initial carrier spreading with a larger diffusion constant[14,15]. To quantify the fast component, we fitted the transients using the model described in Supplementary Section III and extracted the Gaussian width σ. As summarized in Supplementary Figure 6c, σ is slightly smaller for 1035 nm excitation, suggesting somewhat faster hot-carrier cooling. This slight wavelength dependence might be related to the smaller excess energy at 1035 nm, which could allow more efficient cooling, although the detailed microscopic origin remains uncertain and is beyond the scope of this study.

Importantly, while the absolute cooling time shows a modest wavelength dependence, as expected from the different excess carrier energies, the qualitative features that identify the fast component, including its polarity and its picosecond timescale, remain unchanged across wavelengths. We therefore conclude that the mechanism discussed in the main text is not specific to 517 nm excitation but reflects an intrinsic hot-carrier transport process in BP.

As shown in Supplementary Figure 6d, the amplitude of the fast component exhibits a clear saturation behavior as a function of pump power. This behavior is inconsistent with multiphoton absorption or carrier multiplication, both of which typically produce super-linear scaling ($\propto P^2$ or higher for multiphoton absorption, and a stronger-than-linear scaling for carrier multiplication). We therefore attribute the fast component to hot carriers generated by a single-photon absorption process. We fitted the pump-power dependence using a standard saturation model A_{sat} (1 - $\exp^{-P/P_0}$). The fit yields $P_0 = 325 \pm 74$ μW. Since all measurements in the main text were performed at 200 μW (well below P_0), the experiments were carried out in the linear hot-carrier extraction regime.

VII. Simulation of super-diffusion

In this section, we perform a full numerical simulation of super-diffusive transport and directly compare the results with the experimentally measured ultrafast photocurrent

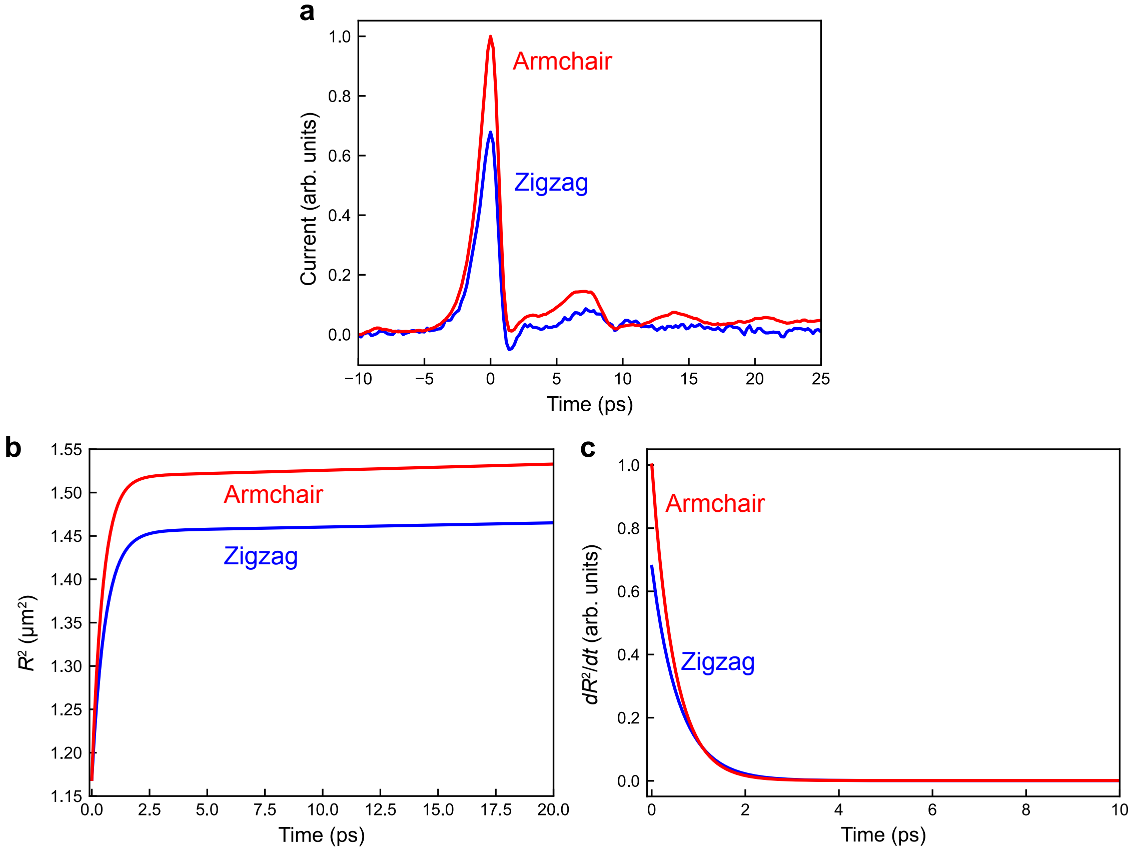

Supplementary Figure 7 | Crystal orientation dependence. **a**, Photocurrent waveforms for the armchair and zigzag directions, normalized to the armchair amplitude. The signals are scaled to account for the responsivity of the photoconductive switch. **b**, Simulated second moment $R^2(t)$ as a function of time after photoexcitation. **c**, Time derivative of $R^2(t)$, used to estimate the photocurrent amplitude at the contact.

(Supplementary Figure 7a).

In the super-diffusive regime, the early-time spreading of photoexcited hot carriers is governed by a transient diffusivity that is strongly enhanced immediately after excitation and rapidly decreases as carriers cool on a sub-picosecond timescale. To model this process, we follow the super-diffusive transport framework introduced by Najafi *et al.*,[16] incorporating the intrinsic in-plane anisotropy of BP together with a time-dependent hot-hole diffusivity. Importantly, in the present calculations, all material parameters except for the hot-carrier enhancement factor are taken directly from the literature.

The diffusivity along each crystallographic direction (AC and ZZ) is taken to follow the same exponentially decaying form used in Ref. 16,

$$D_i(t) = D_{i,\infty} + \left(D_{i,\mathrm{hot}} - D_{i,\infty}\right) \exp\left(-t/t^{i}_{\mathrm{decay}}\right),$$

where i = AC, ZZ. The decay times are fixed to crystallographic-direction–dependent hot-carrier cooling times reported in ultrafast electron diffraction measuremtents[17] with $t^{\mathrm{AC}}_{\mathrm{decay}}$ = 480 fs and $t^{\mathrm{ZZ}}_{\mathrm{decay}}$ = 580 fs. The cold-carrier diffusivity is obtained from the Einstein relation $D_{i,\infty} = \mu_i k_\mathrm{B} T/q$, with the armchair mobility fixed at μ_{AC} = 70 $\mathrm{cm^2/Vs}$ (Fig. 2b) and the zigzag mobility determined from the mobility anisotropy reported in density-functional-theory study[18], yielding μ_{ZZ} = 48 $\mathrm{cm^2/Vs}$. The hot-carrier diffusivities are then defined as $D_{i,\mathrm{hot}} = 1000\, D_{i,\infty}$, which is the only phenomenological assumption introduced in the model and is consistent with the super-diffusion reported in Ref. 16.

The initial carrier distribution is taken as a Gaussian profile reflecting the laser excitation spot,

$$n(r,0) = n_0 \exp\left(-\frac{r^2}{2\sigma_0^2}\right),\ \sigma_0 = \frac{\mathrm{FWHM}}{2\sqrt{2\ln 2}},$$

which preserves its Gaussian form during evolution. The radial expansion is quantified by the second moment,

$$R^2(t) = \frac{\int_0^\infty r^2 n(r,t)\, 2\pi r\, dr}{\int_0^\infty n(r,t)\, 2\pi r\, dr},$$

allowing us to directly track the direction-dependent spreading dynamics.

As shown in Supplementary Figure 7b, at early times the growth of $R^2(t)$ is dominated by the large transient diffusivities $D_{i,\mathrm{hot}}$, resulting in a super-diffusive expansion with a larger value along the armchair direction. In our contact-edge excitation geometry, this early-time expansion is proportional to the number of holes reaching the electrode. Accordingly, the photocurrent amplitude can be estimated from the time derivative dR^2/dt as shown in Supplementary Figure 7c. The relative amplitudes between the AC and ZZ directions show excellent agreement between experiment and simulation. In contrast, the decay times for the AC and ZZ directions are nearly identical, reflecting the similar hot-carrier cooling times ($t^{\mathrm{AC}}_{\mathrm{decay}}$ = 480 fs and $t^{\mathrm{ZZ}}_{\mathrm{decay}}$ = 580 fs). This quantitative agreement in both amplitude anisotropy and decay dynamics provides strong support for the super-diffusive transport scenario.

Supplementary References

1. Akamatsu, T. *et al.* A van der Waals interface that creates in-plane polarization and a spontaneous photovoltaic effect. *Science* **372**, 68–72 (2021).
2. Zeng, Z. *et al.* Dual polarization-enabled ultrafast bulk photovoltaic response in van der Waals heterostructures. *Nat Commun* **15**, 5355 (2024).
3. Zimmermann, P. & Holleitner, A. W. On-site tuning of the carrier lifetime in silicon for on-chip THz circuits using a focused beam of helium ions. *Appl Phys Lett* **116**, 073501 (2020).
4. Wang, Y. *et al.* Ultrafast recovery time and broadband saturable absorption properties of black phosphorus suspension. *Appl Phys Lett* **107**, (2015).
5. Wang, K. *et al.* Ultrafast Nonlinear Excitation Dynamics of Black Phosphorus Nanosheets from Visible to Mid-Infrared. *ACS Nano* **10**, 6923–6932 (2016).
6. Huber, R. *et al.* How many-particle interactions develop after ultrafast excitation of an electron-hole plasma. *Nature* **414**, 286–289 (2001).
7. Zuev, Y. M., Chang, W. & Kim, P. Thermoelectric and Magnetothermoelectric Transport Measurements of Graphene. *Phys Rev Lett* **102**, 096807 (2009).
8. Gabor, N. M. *et al.* Hot Carrier–Assisted Intrinsic Photoresponse in Graphene. *Science* **334**, 648–652 (2011).
9. Yoshioka, K. *et al.* Ultrafast intrinsic optical-to-electrical conversion dynamics in a graphene photodetector. *Nat Photonics* **16**, 718–723 (2022).
10. Song, J. C. W., Rudner, M. S., Marcus, C. M. & Levitov, L. S. Hot Carrier Transport and Photocurrent Response in Graphene. *Nano Lett* **11**, 4688–4692 (2011).
11. Youngblood, N., Chen, C., Koester, S. J. & Li, M. Waveguide-integrated black phosphorus photodetector with high responsivity and low dark current. *Nat Photonics* **9**, 247–252 (2015).
12. Prechtel, L. *et al.* Time-Resolved Photoinduced Thermoelectric and Transport Currents in GaAs Nanowires. *Nano Lett* **12**, 2337–2341 (2012).
13. Buscema, M., Groenendijk, D. J., Steele, G. A., van der Zant, H. S. J. & Castellanos-Gomez, A. Photovoltaic effect in few-layer black phosphorus PN junctions defined by local electrostatic gating. *Nat Commun* **5**, 4651 (2014).
14. Guo, Z. *et al.* Long-range hot-carrier transport in hybrid perovskites visualized by

ultrafast microscopy. *Science* **356**, 59–62 (2017).

15. Fu, S. *et al.* Unveiling high-mobility hot carriers in a two-dimensional conjugated coordination polymer. *Nat Mater* **24**, 1457–1464 (2025).
16. Najafi, E., Ivanov, V., Zewail, A. & Bernardi, M. Super-diffusion of excited carriers in semiconductors. *Nat Commun* **8**, 15177 (2017).
17. Zahn, D. *et al.* Anisotropic nonequilibrium lattice dynamics of black phosphorus. *Nano Lett* **20**, 3728–3733 (2020).
18. Qiao, J., Kong, X., Hu, Z.-X., Yang, F. & Ji, W. High-mobility transport anisotropy and linear dichroism in few-layer black phosphorus. *Nat Commun* **5**, 4475 (2014).
